# Histidine-rich glycoprotein as a prognostic biomarker for sepsis

**DOI:** 10.1038/s41598-021-89555-z

**Published:** 2021-05-13

**Authors:** Kosuke Kuroda, Kenzo Ishii, Yuko Mihara, Naoya Kawanoue, Hidenori Wake, Shuji Mori, Michihiro Yoshida, Masahiro Nishibori, Hiroshi Morimatsu

**Affiliations:** 1 Department of Anesthesiology and Resuscitology, Okayama University Graduate School of Medicine, Dentistry and Pharmaceutical Sciences, 2-5-1 Shikata-cho, Kitaku, Okayama, 700-8558 Japan; 2 Department of Anesthesiology, Fukuyama City Hospital, 5-23-1 Zaocho, Fukuyama, Hiroshima 721-8511 Japan; 3 Department of Pharmacology, Faculty of Medicine, Kindai University, 377-2 Ohnohigashi, Osaka-Sayama, Osaka 589-8511 Japan; 4 Department of Pharmacology, School of Pharmacy, Shujitsu University, 1-6-1 Nishigawara, Nakaku, Okayama, 703-8516 Japan; 5 Center for Innovative Clinical Medicine, Okayama University Hospital, 2-5-1 Shikata-cho, Kitaku, Okayama, 700-8558 Japan; 6 Department of Pharmacology, Okayama University Graduate School of Medicine, Dentistry and Pharmaceutical Sciences, 2-5-1 Shikata-cho, Kitaku, Okayama, 700-8558 Japan

**Keywords:** Diagnostic markers, Predictive markers, Prognostic markers

## Abstract

Various biomarkers have been proposed for sepsis; however, only a few become the standard. We previously reported that plasma histidine-rich glycoprotein (HRG) levels decreased in septic mice, and supplemental infusion of HRG improved survival in mice model of sepsis. Moreover, our previous clinical study demonstrated that HRG levels in septic patients were lower than those in noninfective systemic inflammatory response syndrome patients, and it could be a biomarker for sepsis. In this study, we focused on septic patients and assessed the differences in HRG levels between the non-survivors and survivors. We studied ICU patients newly diagnosed with sepsis. Blood samples were collected within 24 h of ICU admission, and HRG levels were determined using an enzyme-linked immunosorbent assay. Ninety-nine septic patients from 11 institutes in Japan were included. HRG levels were significantly lower in non-survivors (n = 16) than in survivors (n = 83) (median, 15.1 [interquartile ranges, 12.7–16.6] vs. 30.6 [22.1–39.6] µg/ml; p < 0.01). Survival analysis revealed that HRG levels were associated with mortality (hazard ratio 0.79, p < 0.01), and the Harrell C-index (predictive power) for HRG was 0.90. These results suggested that HRG could be a novel prognostic biomarker for sepsis.

## Introduction

Sepsis is one of the leading causes of death worldwide^1,2^. However, all clinical trials conducted for the development of therapeutic drugs for sepsis for decades have failed. One of the problems hindering an early initiation of treatment for sepsis is unavailability of a good biomarker to diagnose and evaluate severity of sepsis^3^. Many markers and scoring systems have been studied to predict the severity and mortality in septic patients. Procalcitonin (PCT) is commonly used to assist in the diagnosis of acute infection in clinical settings^4,5^ and is somewhat considered as a prognostic biomarker^6,7^. However, a single biomarker cannot sufficiently determine the prognosis^3,7,8^. Thus, new biomarkers available to estimate sepsis severity and enable earlier treatment initiation are highly anticipated.

Histidine-rich glycoprotein (HRG) is a 75 kDa plasma glycoprotein produced in the liver and present at concentration of approximately 100 µg/ml^9^. It is considered to be involved in many functions in biological systems, such as coagulation, immune response, and modulation of angiogenesis^9,10^. In particular, HRG is suggested to play an essential role in host defense mechanisms^10–12^. Recently, we reported that plasma HRG levels rapidly decreased in mice with sepsis^13^. Moreover, supplemental HRG infusion significantly improved the sepsis survival rate in mice, while the knockdown of HRG levels exacerbated mortality^13^. Since HRG strongly induced the spherical shape in human neutrophils, suppressed the neutrophil adhesion to vascular endothelial cells, maintained passage through microcapillaries, and reduced ROS production, these effects may contribute to the beneficial effects of HRG detected in septic animals^13^. Furthermore, HRG has been recently reported to protect the vascular endothelial cells from LPS-induced disorder and increased permeability^14^. Our animal study as well as in vitro experiments suggested that plasma HRG might be a useful biomarker of sepsis and that supplemental therapy with HRG may provide a novel strategy for the treatment of sepsis. In fact, our previous clinical study on 70 patients with systemic inflammatory response syndrome (SIRS) showed that HRG levels of septic patients were significantly lower than those of noninfective SIRS patients and that HRG levels were significantly associated with mortality within the SIRS population^15^. However, whether HRG could predict the prognosis of septic patients remains unclear.

This study included septic patients according to Sepsis-2 definition^16^ and evaluate the survival rate of septic patients classified according to plasma HRG levels. HRG levels were also compared with clinical parameters such as PCT in septic patients.

## Results

### Patients

Patients were prospectively enrolled from October 2014 to September 2016 in 11 Japanese hospitals: 2 university and 9 general hospitals. Written informed consent was obtained from 101 patients. Two were excluded because of lack of 28-day survival data, and finally, 99 patients were analyzed. The patient characteristics are shown in Table 1 and Supplementary Tables S1 and S2. The median age was 72.0 (interquartile ranges [IQR], 64.0–78.0) years and 70% were males (69 males and 30 females). Their median acute physiology and chronic evaluation (APACHE) II and sequential organ failure assessment (SOFA) scores were 25.0 (IQR, 21.0–31.0) and 11.0 (IQR, 8.0–13.0), respectively. The 28-day mortality rate was 16.2% (16/99 cases). A total of 46 patients (46.5%) were diagnosed with septic shock, 43 (43.4%) with severe sepsis, and 10 (10.1%) with sepsis according to the Sepsis-2 definition. The 28-day mortality rates in patients with septic shock, severe sepsis, and sepsis were 17.4% (8/46 patients), 18.6% (8/43 patients), 0% (0/10 patients), respectively.

**Table 1:** Patient characteristics.

Variable	Total	Sepsis	Severe sepsis	Septic shock
N	99	10	43	46
Age, years	72.0 (64.0–78.0)	75.0 (68.3–80.5)	72.0 (64.0–80.0)	71.0 (60.8–76.3)
Male sex, n	69 (69.7%)	8 (80.0%)	28 (65.1%)	33 (71.7%)
28-day death, days	16 (16.2%)	0	8 (18.6%)	8 (17.4%)
ICU stay, days	10.0 (5.0–16.0)	7.5 (3.8–15.5)	11.0 (5.0–18.0)	9.5 (5.0–16.0)
APACHE II score	25.0 (21.0–31.0)	20.0 (13.5–21.8)	24.0 (22.0–30.0)	28.0 (21.5–34.5)
SOFA score	11.0 (8.0–13.0)	4.5 (2.0–6.0)	9.0 (7.0–12.0)	13.0 (11.0–16.0)
**Source of infection, n (death)**	99 (16)			
Lung	20 (4)	3	7 (2)	10 (2)
Gastrointestinal	19 (2)	3	10 (1)	6 (1)
Hepatic	1	0	0	1
Gallbladder	8	0	3	5
Urinary	14 (1)	1	6 (1)	7
Bone/soft tissue	13 (2)	2	5 (1)	6 (1)
Others	24 (7)	1	12 (3)	11 (4)
Ventilation, n	53 (53.5%)	5 (50.0%)	23 (53.5%)	25 (54.3%)
Inotropes (Day 1)	54 (54.5%)	1 (10.0%)	7 (16.3%)	46 (100%)
**Blood purification**				
Chronic dialysis	4 (4.0%)	0	1 (2.3%)	3 (6.5%)
Renal replacement therapy	29 (29.3%)	0	15 (34.9%)	14 (30.4%)
Polymyxin B hemoperfusion	7 (7.1%)	0	3 (7.0%)	4 (8.7%)
Liver failure	2 (2.0%)	0	2 (4.7%)	0
AIDS	0	0	0	0
Hematologic malignancies	6 (6.1%)	1 (10.0%)	1 (2.3%)	4 (8.7%)

Expressed as median (interquartile range).

*APACHE* acute physiology and chronic evaluation, *SOFA* sequential organ failure assessment, *AIDS* acquired immunodeficiency syndrome.

### Plasma HRG and PCT levels on the first day of ICU admission

The median HRG level of 99 septic patients was 26.49 (IQR, 19.55–38.40) µg/ml in this study.

**Figure 1:**
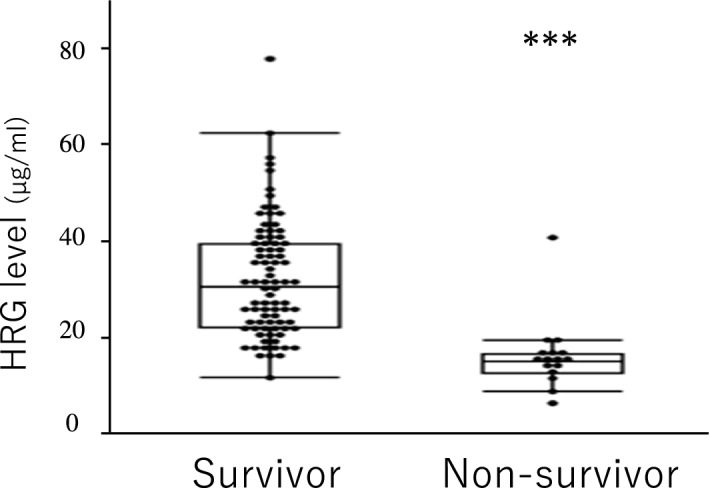
Plasma HRG levels in septic patients on the first day of ICU admission. We compared HRG levels between survivors (n = 83) and non-survivors (n = 16). A box-and-whisker plot showing median, 25th, and 75th percentiles. The bars represent the 5th and 95th percentiles. Circles represent HRG levels in patients. ***P < 0.01. *HRG* histidine-rich glycoprotein.

Figure 1 shows the primary outcome results. Comparing the non-survivors (n = 16) and survivors (n = 83), HRG levels in the former were significantly lower than those in the latter (median, 15.1 [IQR, 12.7–16.6] vs. 30.6 [22.1–39.6] µg/ml; Mann–Whitney test, P < 0.01). PCT levels in non-survivors and survivors were 9.23 (IQR, 3.30–100) and 31.8 (11.0–84.2) ng/ml, respectively, but without statistical differences (P = 0.42).

HRG levels in patients with septic shock, severe sepsis, and sepsis were 25.5 (IQR, 17.6–37.6), 30.8 (19.6–40.9), and 28.9 (22.2–37.8) µg/ml, respectively, but without statistical differences (Kruskal–Wallis test, P = 0.48) (Supplementary Figure S1).

There were no significant correlations between HRG levels and other parameters, such as white blood cell count (Spearman's rho, 0.071, P = 0.48), C-reactive protein (− 0.0018, P = 0.99), PCT (0.089, P = 0.38), platelet number (0.039, P = 0.70), and fibrinogen (0.066, P = 0.55).

### Survival analysis

Table 2 shows associations between plasma levels of each marker and mortality. Higher first-day HRG levels were significantly associated with lower risk of mortality. The hazard ratio (HR) was 0.79 (95% confidence interval [CI], 0.71–0.87, P < 0.01). Even after adjusting with the APACHE II score, the golden standard in evaluating the patient’s severity, the HRG level remained an independent prognostic factor (adjusted HR, 0.81; 95% CI, 0.72–0.89; P < 0.01). PCT levels were not statistically associated with mortality (P = 0.81). The Harrell C-index for mortality was as follows: HRG, 0.90 (95% CI, 0.81–0.99); PCT, 0.54 (0.37–0.71); APACHE II score, 0.72 (0.58–0.86); and SOFA score, 0.68 (0.56–0.81).

**Table 2:** Associations between each variable and mortality.

Variables	Univariate analysis			Adjusted with APACHE II score	
	HR (95% CI)	P	Harrell C-index	Adjusted HR (95% CI)	P
HRG	0.79 (0.71–0.87)	< 0.01	0.90	0.81 (0.72–0.89)	< 0.01
PCT	0.9993 (0.99–1.00)	0.81	0.54	NA	NA
APACHE II score	1.12 (1.06–1.20)	< 0.01	0.72	NA	NA
SOFA score	1.19 (1.04–1.37)	0.010	0.68	NA	NA

We used Cox’s proportional hazard model to evaluate associations between each variable and mortality. *HRG* histidine-rich glycoprotein, *PCT* procalcitonin, *APACHE* Acute Physiology and Chronic Evaluation, *SOFA* Sequential Organ Failure Assessment, *HR* hazard ratio, *Adjusted HR* hazard ratio adjusted according to APACHE II score.

**Figure 2:**
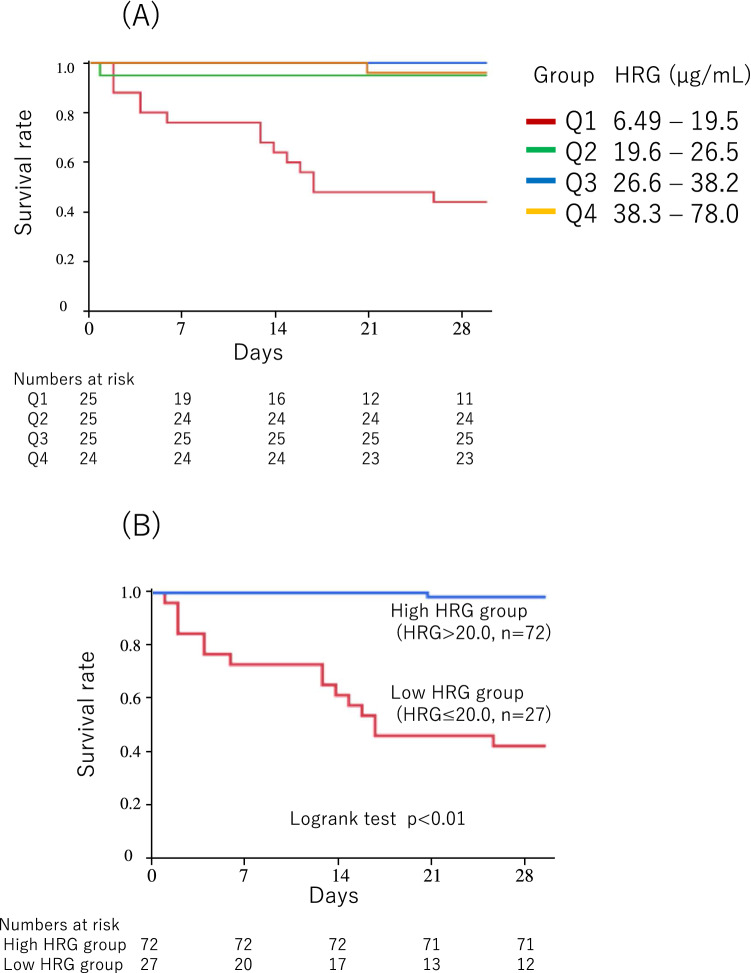
Kaplan–Meier survival curves. (**A**) Patients were divided into four subgroups according to quartiles of HRG level; Q1, 6.49–19.5 µg/ml, n = 25; Q2, 19.6–26.5 µg/ml, n = 25; Q3, 26.6–38.2 µg/ml, n = 25; Q4, 38.3–78.0 µg/ml, n = 24. The mortality of the Q1 group was significantly higher than others (log-rank test, Bonferroni corrected P < 0.01). (**B**) Patients were divided into high and low HRG groups according to the cut-off level of 20.0 µg/ml. At the cut-off level of 20.0 µg/ml, the sensitivity and specificity of HRG levels associated with mortality was 0.94 and 0.84, respectively. *HRG* histidine-rich glycoprotein.

When patients were divided into four subgroups according to quartiles of HRG level, Kaplan–Meier curves (Fig. 2A) showed that the mortality in the Q1 group (lowest HRG subgroup) was significantly higher than that in others (log-rank test, Bonferroni corrected P < 0.01).

Supplementary Table S3 shows that the sensitivity and specificity of HRG levels associated with mortality at the cut-off level of 20.0 µg/ml was 0.94 and 0.84, respectively. Thus, when patients were divided into high and low HRG groups according to this cut-off level, Kaplan–Meier curves (Fig. 2B) showed that the mortality in the latter group was significantly higher than that in the former (HR, 57.2; 95% CI, 11.5–1034; P < 0.01).

We analyzed whether HRG was associated with mortality, regardless of the severity of organ dysfunction. Supplementary Figure S2 shows the associations between HRG level and mortality in subgroups stratified with APACHE II score and each parameter of SOFA score: shock, respiratory dysfunction, liver dysfunction, renal dysfunction, and platelet number. HRG levels were shown to be significantly associated with mortality in all subgroups.

## Discussion

In this multicenter prospective study, first-day HRG levels in non-survivors were demonstrated to be significantly lower than those in survivors among septic patients. HRG levels were also shown to be significantly associated with mortality, and determination of HRG provided a good prognostic estimation as a biomarker for sepsis. Moreover, these properties could also be found in patients with organ failure, such as severe hepatic or renal failure.

In our previous investigation, 70 patients with SIRS, including 20 with sepsis, were examined. We showed that HRG levels in septic patients were significantly lower than those in noninfective SIRS patients and that HRG levels were significantly associated with mortality within the SIRS population^15^. However, the association of HRG with sepsis survival was difficult to determine due to the limited sample size^17^. In the current study, we focused on sepsis and investigated a new cohort of 99 septic patients from 11 institutes, and we could obtain similar results. Thus, these results were reproducible in independent cohorts.

In this study, first-day HRG levels were shown to be associated with mortality. Harrell C-index, the predictive power index, for mortality was 0.90 for HRG. This C-index was superior to the C-indices of APACHE II (0.72) and SOFA scores (0.68), the golden standards in evaluating the sepsis severity. The SOFA score is included in the new definition of sepsis (Sepsis-3)^18^, and previous studies demonstrated that SOFA score is a good indicator of prognosis in ICU-admitted critically ill patients, especially those with severe sepsis^19–21^. Conversely, Liu et al. reported in their meta-analysis that PCT was associated with mortality in septic patients and can moderately predict sepsis mortality^6^. However, the prognostic accuracy of PCT has been reported to be inadequate for clinical use, especially in patients with renal failure^22,23^. In the current study, the C-index for PCT was 0.54 and inferior to that for HRG. Therefore, we suggest that HRG might be superior to APACHE II score, SOFA score, and PCT as a prognostic biomarker.

First-day HRG levels were found to distinguish survivors from non-survivors. Kaplan–Meier curves (Fig. 2A) clearly showed that only Q1 group, patients with first-day HRG levels of < 19.5 µg/ml, had high mortality rate of ≥ 50% and that almost all patients in other groups survived. This unique property would facilitate the determination of death threshold when using HRG as a biomarker. In other words, HRG was thought to identify patients who really need intensive treatments. Intensive and timely care for patients with first-day HRG levels of < 19.5 µg/ml may possibly reduce sepsis mortality. In addition, our previous animal study suggested that supplemental infusion of HRG might provide a novel strategy for the treatment of sepsis^13^, indicating that HRG might serve as a therapeutic as well as a biomarker.

Moreover, when patients were divided into subgroups according to the degree of organ dysfunctions, reduced HRG levels were shown to be significantly associated with mortality in all subgroups, including hepatic and renal dysfunction (Supplementary Figure S2). Current sepsis biomarkers have problems in evaluating patients with organ failure^7^. There are some cases in which PCT cannot work well, especially in patients with renal failure^7,23^. Our data suggested that HRG had sufficient ability in evaluating the severity of septic patients in these settings, including hepatic and renal failures. These results strongly suggested that plasma HRG would be a useful prognostic biomarker for sepsis.

This study had some limitations. First, the old definition of sepsis (Sepsis-2) was used because this study was started before the Sepsis-3 definition was established. Old data could affect interpretation of our results. In this study, all patients had suspicious infection and SOFA score of 2 or higher, however it is unclear whether it was increase of > 2 score. Therefore, another study should be conducted using the Sepsis-3 definition, and we have already started the study. Second, although we focused on sepsis survival, we studied 99 septic patients, including 16 non-survivors. To reinforce our claim that HRG could be a prognostic marker for sepsis, a larger study should be conducted. Third, we had no data on exact number of eligible patients and the incidence rate of sepsis in 11 hospitals. Selection bias could affect the results. Fourth, only one-time HRG levels were assessed on day 1 of ICU, and we had no data on the time course and the exact timing of blood sampling. Timing of sampling in consideration of treatment, and time-dependent changes in HRG levels would be more valuable and may reflect treatment effects. Whether HRG-guided therapy is possible with time course data may be evaluated. Fifth, we had no data regarding patient comorbidities and the pathogen. Further investigations on the association of HRG with patient characteristics and with the causes of sepsis will be conducted. Sixth, because of the lack of exact data on the cause of death, it is possible that patients with withdrawal from treatment were included, which could affect the results. Seventh, for measuring HRG, we used our in-house ELISA, which established in our previously published study. This may affect the results of this study.

In conclusion, in this study, first-day plasma HRG levels in non-survivors were found to be significantly lower than those in survivors among septic patients. HRG were also found to have a good prognostic accuracy as a biomarker for sepsis. Therefore, we suggest that HRG might be a good biomarker in diagnosing sepsis, evaluating sepsis severity, and predicting patient outcomes. Studies with larger cohorts are needed to confirm our findings.

## Methods

### Study design

This was a multicenter, prospective, and observational investigation approved by the Institutional Review Board of any institution involved, including the Okayama University Graduate School of Medicine, Dentistry, and Pharmaceutical Sciences. It was registered at the UMIN Clinical Trials Registry (UMIN000017651, registered 22 May 2015—retrospectively registered, principal investigator's name is Hiroshi Morimatsu). This study was performed in accordance with the ethical standards laid down in the Declaration of Helsinki and its later amendments. This observational study was also reported following the Strengthening the Reporting of Observational Studies in Epidemiology guidelines^24^.

### Patients and data collection

Patients who were newly diagnosed with sepsis according to Sepsis-2^16^ were prospectively enrolled in the study. The inclusion criteria were patients admitted to the ICU and the occurrence of sepsis. The exclusion criteria were age younger than 20 years, pregnancy, or failure to obtain consent.

Clinical and laboratory data were obtained daily while in the ICU. Initial SOFA score and APACHE II score were calculated using clinical parameters and blood test results. Patients were classified according to the previous definitions of sepsis (Sepsis-2), the guidelines of the American College of Chest Physicians/Society of Critical Care Medicine and the International Surviving Sepsis Campaign Guidelines Committee^16^. A follow-up at 28 days was performed to determine survivors and non-survivors.

### Measurement methods

After obtaining written informed consent for participation from the patients or their relatives, blood samples were collected in tubes containing K_2_-EDTA at a convenient timing within 24 h of sepsis diagnosis, regardless of the treatment. The samples were processed and immediately frozen in each hospital, then transported to Okayama University, and stored at − 80 °C for later analysis.

HRG levels were determined using a modified quantitative sandwich enzyme-linked immunosorbent assay (ELISA) as described previously^15^. In brief, we used a rat monoclonal antibody against human HRG (made in-house, number 75-14) as the capture antibody and horseradish peroxidase-conjugated nickel–nitrilotriacetic acid (Ni–NTA HRP conjugate; Qiagen, Venlo, Netherland) for detection instead of secondary antibody. Plasma samples were diluted 1:50 in phosphate-buffered saline (PBS) containing 1% bovine serum albumin (BSA) for measurement. A standard curve is established using serial dilutions of known amounts of purified HRG (made in-house). Each plasma sample was tested in duplicate, and assays were repeated twice independently.

### Outcomes

The primary outcome was HRG levels in survivors and non-survivors. The secondary outcome was the association between HRG levels and mortality.

### Statistical analysis

The statistical approach was mostly designed a priori. Only the analysis provided in Supplementary Figure S2 arose out of the data exploration process and was therefore designed post hoc.

The median, interquartile ranges (IQR, 25th–75th percentiles), and box-whisker plot were used to summarize the variables. The ICU first-day markers were compared using the Mann–Whitney/Kruskal–Wallis tests between/among subgroups, and the statistical significance level was set as 0.05 (two-sided). The contribution of each marker to the 28-day mortality was assessed using Cox’s proportional hazard model adjusted with APACHE II score, also especially in case of HRG, stratified with each parameter of SOFA score. The hazard ratio (HR) and 95% confidence interval (CI) were estimated. The cumulative survival rate was estimated and tested using the Kaplan–Meier method and the log-rank test with Bonferroni correction for four subgroups according to quartiles of HRG level, and using the Kaplan–Meier method and the log-rank test for subgroups defined by the cut-off value of HRG specified with the logistic regression receiver operating characteristic analysis; the HR was calculated using the Cox’s proportional hazard model. Spearman rank correlation coefficient was calculated to assess correlations between HRG and other variables. All analyses were performed using the JMP Pro 12 software (SAS Institute Inc., Chicago, IL), except for the Harrell C-index that was performed using the STATA 12 software (SAS Institute Inc.).

## Supplementary Information

Supplementary Information.

## Data Availability

The datasets generated and analysed during the current study are available from the corresponding author on reasonable request.

## Abbreviations

APACHE: Acute physiology and chronic evaluation
CI: Confidence interval
ELISA: Enzyme-linked immunosorbent assay
HR: Hazard ratio
HRG: Histidine-rich glycoprotein
ICU: Intensive care unit
IQR: Interquartile ranges
PCT: Procalcitonin
SIRS: Systemic inflammatory response syndrome
SOFA: Sequential organ failure assessment
