# Supplementary material for: Histidine-rich glycoprotein as a prognostic biomarker for sepsis
Source: Sci Rep. 2021 May 13;11:10223. doi: 10.1038/s41598-021-89555-z (PMC8119687; doi:10.1038/s41598-021-89555-z)

Histidine-rich glycoprotein as a prognostic biomarker for sepsis

Authors: Kosuke Kuroda, M.D., Ph.D.*[1]; Kenzo Ishii, M.D.[2]; Yuko Mihara, R.N.[1]; Naoya Kawanoue, M.D.[1]; Hidenori Wake, Ph.D.[3]; Shuji Mori, Ph.D.[4]; Michihiro Yoshida, Ph.D.[5]; Masahiro Nishibori, M.D., Ph.D.[6]; Hiroshi Morimatsu, M.D., Ph.D.[1]

Affiliation:

[1]Department of Anesthesiology and Resuscitology, Okayama University Graduate School of Medicine, Dentistry and Pharmaceutical Sciences, Okayama, Japan

[2]Department of Anesthesiology, Fukuyama City Hospital, Fukuyama, Japan

[3]Department of Pharmacology, Faculty of Medicine, Kindai University, Osaka, Japan

[4]Department of Pharmacology, School of Pharmacy, Shujitsu University, Okayama, Japan

[5]Center for Innovative Clinical Medicine, Okayama University Hospital, Okayama, Japan

[6]Department of Pharmacology, Okayama University Graduate School of Medicine, Dentistry and Pharmaceutical Sciences, Okayama, Japan

Supplementary Table S1. Patient characteristics

Biochemical data (Day 1). Expressed as median (interquartile range). WBC, white blood cell count; Ht, hematocrit; Plt, platelet number; CRP, C-reactive protein; BUN, blood urea nitrogen; Cr, creatinine; T. bil, total bilirubin; AST, aspartate aminotransferase; Alb, albumin; Fbg, fibrinogen; Lac, lactate; PCT, procalcitonin.

Variable	Total	Sepsis	Severe sepsis	Septic shock
WBC (/L)	9,200 (3,100-16,170)	10,700 (4,620-15,620)	6,990 (3,200-16,170)	9,280 (2,370-17,620)
Ht (%)	28.0 (22.9-31.6)	27.2 (23.7-30.3)	26.9 (22.7-31.0)	28.5 (23.0-33.2)
Plt (*10000/L)	6.6 (1.7-14.9)	18.5 (13.9-24.8)	6.1 (2.5-10.2)	4.9 (0.9-11.7)
CRP (mg/dL)	16.2 (9.9-23.3)	10.6 (7.1-23.5)	16.3 (9.3-23.1)	16.5 (11.9-23.4)
BUN (mg/dL)	33.5 (20.8-44.6)	19.6 (14.8-25.0)	37.3 (23.3-48.9)	33.3 (21.7-49.3)
Cr (mg/dL)	1.4 (1.1-2.6)	0.9 (0.6-1.2)	1.4 (1.1-2.6)	1.6 (1.1-3.2)
T. bil (mg/dL)	1.1 (0.7-2.0)	0.8 (0.5-1.0)	1.1 (0.8-1.9)	1.5 (0.7-2.3)
AST (U/L)	49.0 (24.0-105.0)	27.0 (19.5-85.5)	37.0 (20.0-99.0)	68.5 (32.8-149.0)
Total protein (g/dL)	5.0 (4.3-5.8)	5.3 (4.7-5.9)	4.9 (4.6-5.6)	5.2 (3.9-5.9)
Alb (g/dL)	2.4 (2.0-2.8)	2.5 (2.2-2.9)	2.6 (2.1-2.9)	2.3 (1.9-2.7)
Fbg (mg/dL)	423.0 (281.3-563.0)	407.0 (365.0-463.0)	453.5 (281.3-671.0)	419.0 (259.5-549.0)
Lac (mmol/L)	3.3 (2.0-5.1)	3.0 (1.3-4.5)	2.5 (1.7-3.8)	4.5 (2.6-8.5)
PCT (ng/mL)	31.6 (8.5-91.9)	17.4 (6.9-53.4)	20.7 (6.9-83.2)	34.7 (14.4-100.0)

Supplementary Table S2. Patient characteristics of survivors and non-survivors

Expressed as median (interquartile range). APACHE, Acute Physiology and Chronic Evaluation; SOFA, Sequential Organ Failure Assessment; AIDS, acquired immunodeficiency syndrome; WBC, white blood cell count; CRP, C-reactive protein; PCT, procalcitonin; Lac, lactate.

Variable	Total	Survivors	Non-survivors
N	99	83	16
Age, years	72.0 (64.0-78.0)	72.0 (64.0-78.0)	72.0 (62.0-81.0)
Male sex, n	69 (69.7%)	58 (69.9%)	11 (68.8%)
ICU stay, days	10.0 (5.0-16.0)	10.0 (5.0-18.0)	6.0 (2.0-15.0)
APACHE II score	25.0 (21.0-31.0)	24.0 (20.0-29.0)	32.0 (24.0-42.0)
SOFA score	11.0 (8.0-13.0)	10.0 (7.0-13.0)	12.0 (11.0-17.0)
Source of infection, n			
Lung	20	16	4
Gastrointestinal	19	17	2
Hepatic	1	1	0
Gallbladder	8	8	0
Urinary	14	13	1
Bone / soft tissue	13	11	2
Others	24	17	7
Ventilation, n	53 (53.5%)	39 (47.0%)	14 (87.5%)
Inotropes (Day 1)	54 (54.5%)	45 (54.2%)	9 (56.3%)
Blood purification			
Chronic dialysis	4 (4.0%)	4 (4.8%)	0
Renal replacement therapy	29 (29.3%)	22 (26.5%)	7 (43.8%)
Polymyxin B hemoperfusion	7 (7.1%)	6 (7.2%)	1 (6.3%)
Liver failure	2 (2.0%)	2 (2.4%)	0
AIDS	0	0	0
Hematologic malignancies	6 (6.1%)	6 (7.2%)	0
Biochemical data (Day 1)			
WBC, /L	9,200 (3,100-16,100)	9,200 (3,500-16,100)	8,100 (1,100-18,300)
CRP, mg/mL	16.2 (9.9-23.3)	16.2 (10.1-23.5)	16.0 (8.8-22.0)
PCT, ng/mL	31.6 (8.5-91.9)	31.8 (11.0-84.2)	9.2 (3.2-100.0)
Lac, ng/mL	3.3 (2.0-5.1)	3.0 (1.8-4.9)	4.1 (2.6-13.1)

Supplementary Table S3. Significance of HRG for predicting mortality

PPV and NPM denote positive and negative predictive values, respectively.

Cut off values (μg/mL)	Sensitivity	Specificity	PPV	NPV
10.0	0.19	1.00	1.00	0.86
15.0	0.56	0.99	0.90	0.92
20.0	0.94	0.84	0.54	0.99
25.0	0.94	0.64	0.34	0.98
30.0	0.94	0.49	0.26	0.98

Supplementary Figure S1

Plasma HRG levels on the first day in subgroups.

HRG levels in patients with septic shock (n = 46), severe sepsis (n = 43), and sepsis (n = 10) were compared, but without statistical differences (Kruskal–Wallis test, P = 0.48). A box-and-whisker plot showing median, 25th, and 75th percentiles. The bars represent the 5th and 95th percentiles. Open and filled circles represent HRG levels in survivors and non-survivors, respectively. HRG denotes histidine-rich glycoprotein.

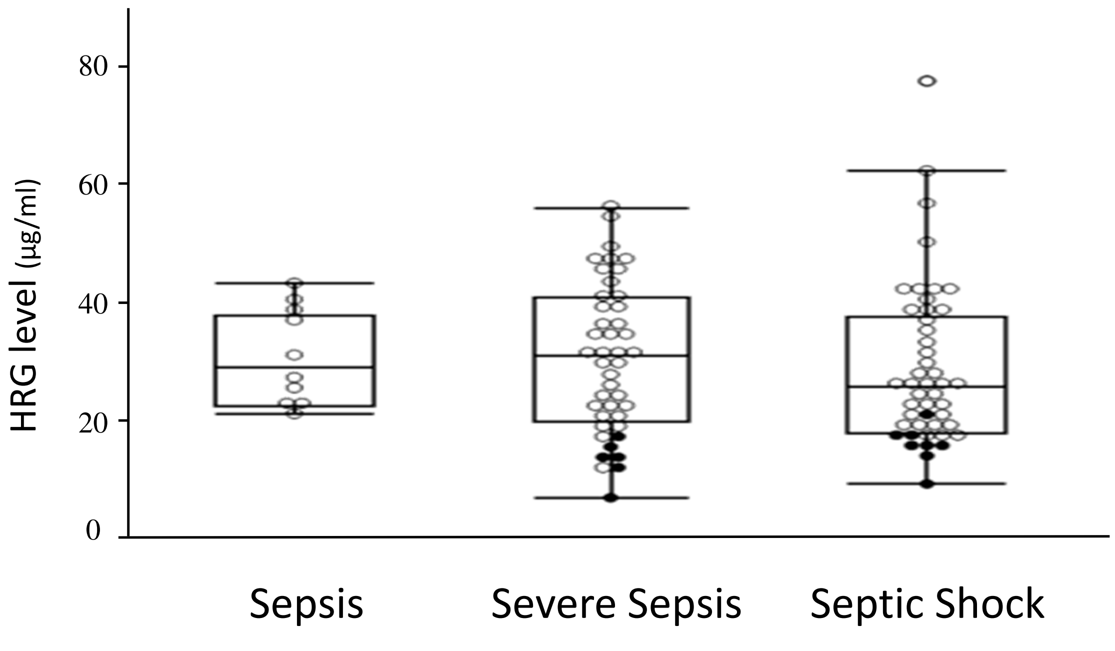

Supplementary Figure S2

Associations between HRG levels and mortality in subgroups.

The hazard ratio was calculated using the Cox's proportional hazard model. Significant associations were found between HRG levels and mortality in all subgroups: patients with and without high APACHE II score, shock, respiratory dysfunction, liver dysfunction, renal dysfunction, and low platelet number. The hazard ratio was calculated using the Cox's proportional hazard model. APACHE denotes Acute Physiology and Chronic Evaluation. HRG denotes histidine-rich glycoprotein.

Variable		n	Hazard Ratio (95%CI)
APACHE II score	<25	(n=48)	0.87 (0.76 – 0.97)
	≥25	(n=51)	0.75 (0.64 – 0.85)
Shock	−	(n=53)	0.82 (0.72 – 0.91)
	+	(n=46)	0.73 (0.60 – 0.86)
PaO_2/FiO_2	> 200	(n=48)	0.77 (0.65 – 0.88)
	≤ 200	(n=51)	0.81 (0.70 – 0.92)
Bilirubin	< 2.0	(n=73)	0.81 (0.72 – 0.90)
	≥ 2.0	(n=26)	0.63 (0.39 – 0.86)
Creatinine	< 3.5	(n=83)	0.80 (0.72 – 0.88)
	≥ 3.5	(n=16)	0.61 (0.19 – 0.91)
Platelet	> 50000	(n=57)	0.90 (0.76 – 0.99)
	≤ 50000	(n=42)	0.75 (0.64 – 0.85)

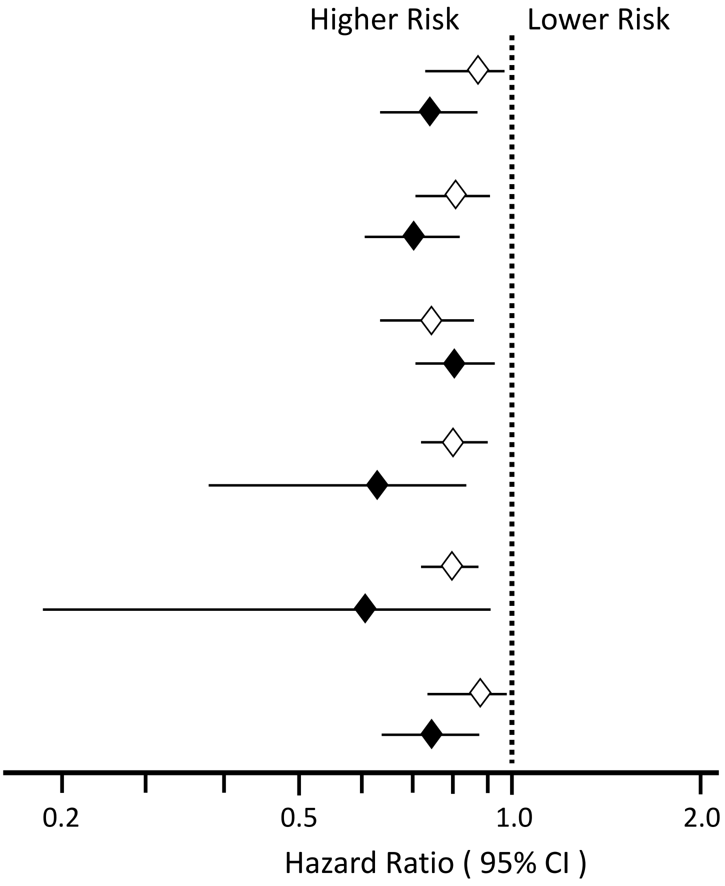

Supplement: Supplementary file 1 — Supplementary Information. [file 41598_2021_89555_MOESM1_ESM.pdf]
